# Acupuncture for menopausal vasomotor symptoms: study protocol for a randomised controlled trial

**DOI:** 10.1186/1745-6215-15-224

**Published:** 2014-06-12

**Authors:** Marie Pirotta, Carolyn Ee, Helena Teede, Patty Chondros, Simon French, Stephen Myers, Charlie Xue

**Affiliations:** 1 Department of General Practice, University of Melbourne, Australia 200 Berkeley St, Carlton, Victoria 3053, Australia; 2 Monash Centre for Health Research and Implementation, School of Public Health and Preventive Medicine, Monash University, Australia Diabetes and Vascular Medicine Unit, Monash Health, Wellington Rd, Clayton VIC 3800, Australia; 3 Canada and Faculty of Health Sciences, School of Rehabilitation Therapy, Queen’s University, Ontario, 99 University Ave, Kingston, ON K7L 3N6, Canada; 4 NatMed-Research Unit, Division of Research, Southern Cross University, Military Road, East Lismore NSW 2480, Australia; 5 School of Health Sciences, RMIT University, 124 Little La Trobe St, Melbourne VIC 3000, Australia

**Keywords:** Acupuncture, Complementary medicine, Menopause, Hot flashes, Vasomotor symptoms, Quality of life, Placebo

## Abstract

**Background:**

Hot flushes and night sweats (vasomotor symptoms) are common menopausal symptoms, often causing distress, sleep deprivation and reduced quality of life. Although hormone replacement therapy is an effective treatment, there are concerns about serious adverse events. Non-hormonal pharmacological therapies are less effective and can also cause adverse effects. Complementary therapies, including acupuncture, are commonly used for menopausal vasomotor symptoms. While the evidence for the effectiveness of acupuncture in treating vasomotor symptoms is inconclusive, acupuncture has a low risk of adverse effects, and two small studies suggest it may be more effective than non-insertive sham acupuncture. Our objective is to assess the efficacy of needle acupuncture in improving hot flush severity and frequency in menopausal women. Our current study design is informed by methods tested in a pilot study.

**Methods/design:**

This is a stratified, parallel, randomised sham-controlled trial with equal allocation of participants to two trial groups. We are recruiting 360 menopausal women experiencing a minimum average of seven moderate hot flushes a day over a seven-day period and who meet diagnostic criteria for the Traditional Chinese Medicine diagnosis of Kidney *Yin* deficiency. Exclusion criteria include breast cancer, surgical menopause, and current hormone replacement therapy use. Eligible women are randomised to receive either true needle acupuncture or sham acupuncture with non-insertive (blunt) needles for ten treatments over eight weeks. Participants are blinded to treatment allocation. Interventions are provided by Chinese medicine acupuncturists who have received specific training on trial procedures. The primary outcome measure is hot flush score, assessed using the validated Hot Flush Diary. Secondary outcome measures include health-related quality of life, anxiety and depression symptoms, credibility of the sham treatment, expectancy and beliefs about acupuncture, and adverse events. Participants will be analysed in the groups in which they were randomised using an intention-to-treat analysis strategy.

**Discussion:**

Results from this trial will significantly add to the current body of evidence on the role of acupuncture for vasomotor symptoms. If found to be effective and safe, acupuncture will be a valuable additional treatment option for women who experience menopausal vasomotor symptoms.

**Trial registration:**

Australian New Zealand Clinical Trials Registry ACTRN12611000393954 11/02/2009.

## Background

### Vasomotor symptoms

Three quarters of menopausal women experience vasomotor symptoms (VMS), or hot flushes and night sweats [1]. VMS last an average of 5.2 years [2] but persist in 10% of women for 15 years or longer [3], and can cause significantly lowered quality of life due to social embarrassment, sleep deprivation, and physical discomfort [4]. Risk factors for VMS include Caucasian ethnicity [5], increased body mass index (BMI) [6,7], being a smoker [3], feeling stressed [8], financial impoverishment [5], and history of depression [8].

### VMS management

Hormone replacement therapy (HRT) is a highly effective treatment for hot flushes, reducing incidence by up to 90% [4]. However, HRT also increases the risk of thromboembolic disease [9], stroke [10], breast cancer [11] and possibly, dementia [12]. Despite growing interest in non-hormonal pharmacological therapies such as psychotropic drugs and selective serotonin reuptake inhibitors (SSRIs) and serotonin-noradrenaline reuptake inhibitors (SNRIs), these are not as effective as HRT, and often cause troublesome adverse events [4,13,14]. Current evidence does not support the use of most complementary medicines (CM) for VMS, with popular treatments such as black cohosh shown to be ineffective [15,16] and only modest benefits reported with soy [17]. Yet, over half of the women surveyed in a 2006 Sydney study reported using CM for their menopausal symptoms, with acupuncturists being the second most popular therapists visited [18].

### Acupuncture

Acupuncture is a form of Chinese medicine that involves the insertion of fine needles into specific points on the surface of body known as acupuncture points, or acupoints [19]. Acupuncture has an excellent safety profile when practised by qualified acupuncturists, with large prospective studies reporting that 90% of patients do not experience any adverse events, and that serious events are rare [20,21].

Some progress has been made on understanding the neural mechanisms of acupuncture. Acupuncture analgesia is modulated by various transmitters, notably endogenous opioids, serotonin and noradrenaline [22,23]. Acupuncture activates the ‘pain matrix’ - areas of the brain that have been shown to be consistently activated by noxious stimuli [24] - which includes the insula, an area that may be involved in the hot flush mechanism [25,26].

### Acupuncture for VMS

The thermoneutral zone is the tolerable temperature zone of the immediate environment within which changes in core body temperature do not cause compensatory sweating or flushing [13]. Researchers propose that low serotonin levels and subsequent high noradrenaline levels during menopause narrow the thermoneutral zone in women experiencing VMS. Treatments that raise central serotonin levels, such as SSRIs or acupuncture, may reduce hot flushes by normalising the thermoneutral zone [4].

Two systematic reviews, published in 2009, found no evidence that acupuncture is effective for VMS, and recommended more rigorous research [27,28]. Since then, four new clinical trials have published their results [29-32]. Two of these were pragmatic randomised trials and reported reductions of mean hot flush scores by 48 to 66% in treatment groups, compared with 28 to 29% in usual care groups (total n = 451) [29,33]. However, a contentious issue in acupuncture research is the use of an adequate sham method in order to control for non-specific effects of acupuncture. One approach is insertive sham acupuncture, which may involve needling of acupuncture points that are considered to be ‘irrelevant’ in the treatment of the condition in question, needling non-acupuncture points, or superficial needling without eliciting a needle sensation. Insertive sham controls are increasingly considered to be an inferior acupuncture control method, with many large randomised controlled trials (RCTs) failing to show a difference between true and insertive sham acupuncture [34]. Five such trials for VMS all failed to demonstrate a difference in mean hot flush scores between groups receiving true and insertive sham acupuncture [35-39]. Alternatively, non-insertive sham acupuncture controls for needling while simulating a needle-prick sensation using a blunt needle which does not penetrate the skin [40,41]. These sham needles have been designed to shorten and ‘telescope’ into themselves, and have been validated as plausible simulations of acupuncture in several studies [41-45]. Three small trials reported true acupuncture to be more effective than non-insertive sham, with greater reductions in VMS frequency [32] and severity [30,46]. Two of these trials were published after the 2009 systematic reviews were conducted [30,32].

Collectively, the extant literature suggests that acupuncture treatment as practised in a community setting can relieve the burden of hot flushes and that the insertion of a needle may represent part of the specific effect of acupuncture on VMS.

### Pilot study of acupuncture for VMS

In 2009, CE, MP and CX undertook a pilot project to assess feasibility of a trial of needle acupuncture compared with non-insertive sham needle for VMS. Twenty-seven women were randomised, and 20 completed the study. Study outcomes and participant feedback were used to modify the design of this current RCT protocol. Our method was feasible and acceptable to participants (results not published).

### Summary

There is a need for safe and effective treatment of menopausal VMS, given the negative impact that hot flushes can have on quality of life. Acupuncture is a popular treatment amongst midlife women and has a favourable safety profile. However, evidence for its effectiveness in treating VMS remains inconclusive, although results from pragmatic studies and studies using non-insertive sham needles are promising. We have used findings from our feasibility study to inform the design of an adequately powered randomised sham-controlled trial using the non-insertive sham needle.

### Aims and hypotheses

Our primary aim is to assess the efficacy of needle acupuncture in improving hot flush severity and frequency in menopausal women. Our hypothesis is that needle acupuncture will result in greater improvement in the severity and frequency of hot flushes compared to non-insertive sham acupuncture after ten treatments given over an eight-week period.

## Methods

### Trial design

The trial, also known as the ‘Acupause’ trial, is a stratified, single (participant)-blind, parallel, randomised sham-controlled trial with equal allocation. The clinical trial results will be reported according to the CONSORT guidelines and the Standards for Reporting Interventions in Clinical Trials of Acupuncture (STRICTA) guidelines [47,48].

### Trial setting

The trial is taking place in 15 private clinics of project acupuncturists (Chinese medicine practitioners) in Australia, located in metropolitan Melbourne, and regional Victoria, New South Wales, and the Gold Coast, Queensland, between September 2011 and October 2014.

The trial has been approved by the Human Research Ethics Committees of the University of Melbourne (1135293 16/6/2011); Monash University (2011001242); RMIT University (1135293); and Southern Cross University (ECN-11-192). It has been registered with the Australian New Zealand Clinical Trial Registry (ACTRN12611000393954) and is funded by a Project Grant from the National Health and Medical Research Council, Australia (APP 1004406).

### Participants

A total of 360 women in the late menopausal transition or postmenopause, who are experiencing hot flushes, are being recruited. A number of recruitment methods are used including (i) advertising through social media (Facebook), University staff and student newsletters, and a health register in a Melbourne tabloid newspaper; (ii) flyers at general practitioner, menopause outpatient and allied health surgeries and female-only fitness centres; (iii) various strategies through Jean Hailes for Women’s Health (http://www.jeanhailes.org.au), an Australian national not-for-profit education and research organisation focusing on women’s health (strategies include utilisation of the organisation’s research register, media releases, social networking connections, website features, and features in consumer and professional newsletters); and (iv) media exposure (radio, television and print).

#### Inclusion criteria

We include women if they:

1. Are deemed postmenopausal (at least 12 months past the final menstrual period) or in the late menopausal transition (Follicular Stimulating Hormone/FSH level of 25 IU or greater, amenorrhoea of ≥ 60 days and currently experiencing VMS) [49]. (Women who have had a hysterectomy are included if an FSH level is greater than 25 IU and they are 51 years of age or older); and

2. Record a mean hot flush score of at least 14 over 7 days during the run-in period (equivalent to an average of seven moderately-severe hot flushes a day and assessed using a validated Hot Flush Diary) [50,51]; and

3. Meet the criteria for the Traditional Chinese medicine (TCM) diagnosis of Kidney *Yin* deficiency determined using a structured Chinese medicine history and examination [52]. Women are included if they score higher for Kidney *Yin* deficiency than for Kidney *Yang* deficiency. See Table 1 for details of the standardised history and examination used.

**Table 1:** Standardised Traditional Chinese Medicine (TCM) history and examination used in the Acupause study

	**Kidney *Yin *** **deficiency scale**	**Kidney *Yang *** **deficiency scale**
History	Sensations of heat in the body with sweating^a^	Cold limbs^a^
		Dizziness or vertigo
	Feelings of heat in the palms, soles and chest	Ache and soreness in the lower back
	Dry mouth or dry hard stool	Frequency of micturition
	Aching and soreness in the lower back and knees Dizziness or tinnitus	Low energy and pale complexion
Examination	Red tongue with scant coat	Pale tongue with thin coat
	Rapid fine pulse	Sunken, fine pulse without force

Each symptom is scored on a 4-point Likert scale for both frequency and severity in the previous month. A score of 1 is given for each tongue and pulse examination that meets the stated criteria.

^a^These symptoms receive a double score as they are considered cardinal symptoms.

Kidney *Yin* deficiency is the most common diagnosis made by TCM practitioners in menopausal women who present with hot flushes [53,54]. We use standardised TCM diagnosis as an inclusion criterion for several reasons. In clinical practice it is usual for an acupuncturist to tailor an acupuncture point prescription to fit the diagnosis that is made after history and examination. However, individualisation of the acupuncture point prescription did not impact on treatment outcome in one pragmatic trial on acupuncture for VMS [54]. By standardising this TCM diagnostic process, we are creating a homogenous sample according to TCM diagnosis, simplifying the treatment protocol, and ensuring our findings are applicable to both TCM and western medical settings.

#### Exclusion criteria

Women are excluded if they:

1. Are younger than 40 years of age (therefore diagnosed as having Premature Ovarian Failure) or have previously been diagnosed with premature ovarian failure and are less than 50 years of age;

2. Have had a bilateral salpingo-oophorectomy;

3. Have any medical reason to be amenorrheic (for example pregnancy, hyperprolactinemia, Cushing’s syndrome);

4. Have poorly controlled hyperthyroidism or hypothyroidism;

5. Are experiencing VMS that started or became worse after diagnosis of breast cancer or commencing treatment for breast cancer;

6. Are currently taking HRT, with the following washout periods to be observed: transdermal (four weeks), oral or intrauterine HRT (eight weeks), progestin implant/oestrogen injectable, phytoestrogen therapy (three months), oestrogen pellet/progestin injectable (six months);

7. Have ceased any other treatment for hot flushes within twelve weeks of screening (including complementary medicines);

8. Have commenced using any other pharmacological or complementary therapy for hot flushes in the past eight weeks;

9. Have commenced using vaginal oestrogen therapy in the past twelve weeks;

10. Are unable to read or write sufficiently in English to complete the outcome measures, which require a Grade 6 reading level;

11. Have had needle acupuncture treatment in the past two years;

12. Have relative contraindications to acupuncture (use of anticoagulant drugs, heart valve disease, poorly controlled diabetes mellitus);

13. Are unwilling or unable to attend for acupuncture/sham acupuncture for ten treatments over eight weeks; or

14. Are unable to give informed consent.

#### Changes to eligibility criteria after trial commencement

To facilitate recruitment within the funded study period, two changes were made to the original eligibility criteria. Originally, any previous acupuncture experience was an exclusion criterion. From October 2011, participants with previous acupuncture experience were deemed to be eligible, as long as the last needle acupuncture treatment was more than two years previously. Use of the Park Sham Device has been validated in non-acupuncture naïve participants [43,55], and advice from the manufacturer was that broadening the inclusion criteria to non-acupuncture naïve participants was reasonable (personal communication, Dr Jongbae Park). Setting a two-year period since a participant’s last acupuncture experience was considered a long enough time frame to minimise familiarity to acupuncture practice.

In July 2012, in the light of emerging evidence and recommendations from the Stages of Reproductive Ageing (STRAW + 10) Workshop (Harlow, Gass *et al*. 2012), we expanded our criteria to include women in the late menopausal transition (a phase now known to be characterised by oestrogen deficiency and high risk of VMS), assessed using FSH testing.

### Trial procedure

**Figure 1:**
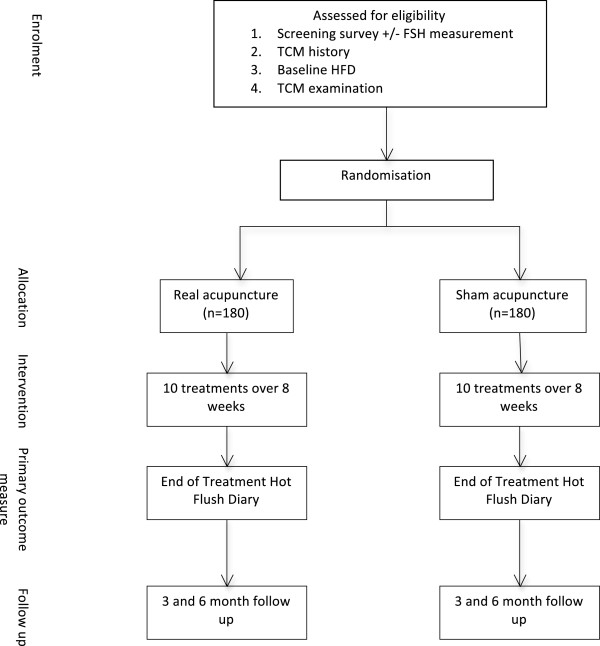
**Flowchart of trial procedures.** FSH = Follicular Stimulating Hormone; TCM = Traditional Chinese Medicine; HFD = Hot Flush Diary.

See Figure 1 for a flowchart of the trial procedures. Potential participants complete an initial screening survey, followed by a TCM questionnaire online or over the telephone with a trained investigator (KN). All details are stored online in a password-protected survey management software account. Women who score higher on the Kidney *Yin* scale than on the Kidney *Yang* scale are sent a baseline Hot Flush Diary (HFD) to complete.

Women recording an average of seven moderately-severe hot flushes a day over the seven- day period are then assessed for TCM criteria (tongue and pulse diagnosis) by a trained acupuncturist (either CE or a project acupuncturist). For quality assurance purposes, the first twenty potential participants’ tongues assessed are photographed after obtaining written consent and an expert Chinese medicine practitioner and researcher (CX) views the de-identified photographs to reconcile the diagnosis with the clinical picture. Thereafter, photographs are only taken of participants if their TCM diagnosis is unclear. Prior to randomisation, women are requested to provide written informed consent after discussing trial procedures and risks of acupuncture with a trained acupuncture researcher (CE/JS).

### Interventions

#### Providers and standardisation of the intervention

Fifteen project acupuncturists at 15 sites administer the study interventions. All acupuncturists have obtained a Bachelor degree in Chinese medicine, have five or more years of clinical experience and are registered with the Chinese Medicine Board of the Australian Health Practitioner Regulation Agency.

To optimise fidelity of delivery of the intervention, project acupuncturists attend a two-hour training session delivered by the acupuncturist researcher (CE) who developed and administered the interventions during the pilot trial. Acupuncturists receive a detailed training manual and a DVD demonstrating the use of the Park Sham Device (sham needle). The training session includes an introduction to basic clinical research methods and a practical demonstration of the Park Sham Device, during which acupuncturists gain ‘hands-on’ experience on how to use the sham needle.

Either the acupuncturist researcher (CE) or a trained non-acupuncturist investigator (SM) in NSW and Queensland, visits project acupuncturists within a few weeks of their first study participant session to ensure protocol adherence. During this visit, a treatment is observed and feedback provided to the acupuncturist. De-identified photographs of the needling at interstate training sessions are Emailed to CE so that point location could be assessed.

Acupuncturists record details of each treatment on individual Case Report Forms, which are kept in a locked cupboard in the clinic and returned to the research team every six months.

#### Treatment rationale

The standardised treatment protocol is based on TCM principles and is designed to treat Kidney *Yin* deficiency. The protocol was developed by consensus between two practising acupuncturist researchers (CX and CE) after considering a textbook, literature review and expert opinions from three leading international acupuncture researchers with particular expertise in women’s health.

While the number of points for an adequate ‘dose’ of acupuncture is yet to be determined [56], this current study protocol follows the minimum protocols used in other positive studies of acupuncture for hot flushes. In particular the positive study by Nir *et al*. used an average of six points, and nine treatments were provided [46]. A literature review suggests that at least ten treatments should be provided for hot flush treatment [57]. The acupuncture points chosen for this trial are included in the group of eight core acupuncture points that were used in one pragmatic trial [54] which allowed for individualisation of the treatment protocol by acupuncturists. However, no relation was found between choice of particular acupuncture points and treatment outcome in that study, suggesting that point specificity may not be of relevance when using acupuncture to treat hot flushes.

#### Treatment regimen

All participants receive ten thirty-minute treatments over eight weeks, twice weekly in the first two weeks and weekly thereafter. Treatments are provided at no cost to participants. All attempts are made to minimise missed sessions and limit breaks between sessions to two weeks or less.

#### True acupuncture

True acupuncture needles are standard stainless steel, sterile and disposable, 32-gauge in thickness and 40 mm in length. Table 2 describes the acupuncture point prescription. A standardised prescription of six acupuncture points is used unilaterally. Needles are inserted and manipulated manually until needling sensation (*de qi)* is obtained, and are retained for 20 [56] minutes with manual manipulation at 10 minutes. *De qi*, literally meaning ‘arrival of energy’, is a term used in acupuncture and refers to a sensation of numbness or distension sometimes generated by stimulating acupuncture needles. According to acupuncture theory, activation of *de qi* may be one indication that acupuncture is exerting its beneficial effects [56]. Acupuncturists are trained to enquire about specific needle sensations when providing true acupuncture.

**Table 2:** Acupoints used in the true acupuncture group in the Acupause study (Unilateral)

**Acupoint (standard abbreviation/Chinese nomenclature)**	**Location**	**Indication**	**Depth of insertion**
Kidney 6 (KI6/*Zhaohai*)	In the depression below the tip of the medial malleolus	Tonifies Kidney *Yin*	up to 3 mm
Kidney 7 (KI7/*Fuliu*)	2 *cun*^a^ directly above the acupoint Kidney 3 on the anterior border of the Achilles tendon. (Kidney 3 is located in the depression between the tip of the medial malleolus and the Achilles tendon)	Tonifies Kidney *Yang* and stops night sweating	up to 15 mm
Spleen 6 (SP6/*Sanyinjiao*)	3 *cun* directly above the tip of the medial malleolus	Nourishes Kidney, Heart and Liver *Yin*	up to 20 mm
Heart 6 (HT6/*Yinxi*)	When the palm faces upward, the point is on the radial side of the tendon of muscularis flexor carpi ulnaris, 0.5 *cun* above the transverse crease of the wrist	Together with KI7, stops night sweating	up to 3 mm
Conception Vessel 4 (CV4/*Guanyuan*)	On the anterior midline, 3 *cun* below the umbilicus	Strengthens the uterus, nourishes the kidneys	20 to 30 mm
Liver 3 (LR3/*Taichong*)	On the dorsum of the foot, in the depression distal to the junction of the first and second metatarsal bones	Subdues rising Liver *Yang*	7 to 12 mm

^a^A *cun* is is a measurement used in locating acupoints, and corresponds to the distance between the two medial ends of the creases of the interphalangeal joints, when the patient’s middle finger is flexed.

#### Sham acupuncture

**Figure 2:**
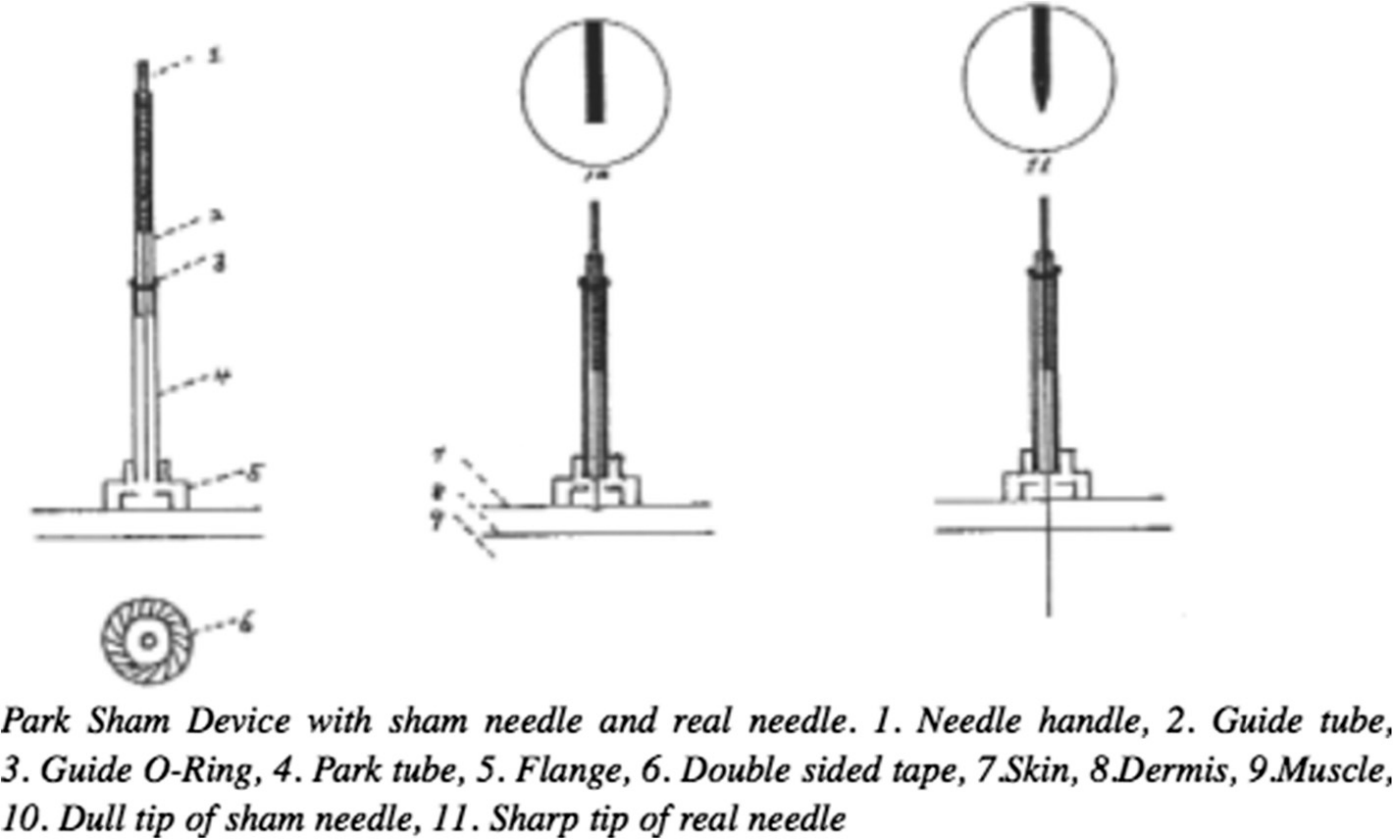
**Diagram of Park Sham device.** Reproduced from Acupuncture in Medicine, Jongbae Park, Adrian White, Clare Stevinson, Edzard Ernst, Martin James, vol 20, p 168-174, copyright 2002 with permission from BMJ Publishing Group Ltd.

We use a non-insertive sham control, the Park Sham Needle, which is supported by a base unit consisting of a plastic ring and guide tube and attached to the skin with double-sided tape. The needle and base unit is collectively referred to as the Park Sham Device (see Figure 2). This sham needle has been validated in both acupuncture-naïve and acupuncture-experienced participants, and in healthy volunteers and patients [41,43,44,55,58]. It has been demonstrated to be less likely to induce *de qi* or the specific needling sensation than a true acupuncture needle [41].

Participants in the control group receive bilateral ‘needling’ on three non-acupuncture points which are not located on the same neuromuscular segments as the prescribed points used in the true acupuncture group, so as to minimise any segmental effects. Table 3 describes the sham points used. All other aspects of the sham intervention (use of the Park Sham Device base unit, needle retention time and manipulation, asking about needle sensation, treatment schedule) are as described above for the true acupuncture group.

**Table 3:** Points used in the sham acupuncture group in the Acupause study (bilateral)

**Name given to point**	**Location**	**Relationship to meridians and acupoints**	**Innervation**
Abd 1	2 *cun*^a^ above and 5 *cun* lateral to the umbilicus	1 *cun* lateral to the Spleen meridian	T8/9
Arm 1	Midway between the acupoints Lung 5 and Large intestine 11 on the cubital crease		C5/6
Thigh 1	On the bulge of the rectus femoris, 5 *cun* above the middle of the superior border of the patella	2 *cun* lateral and 3 *cun* proximal to real acupoint Spleen 10	L3

^a^A *cun* is is a measurement used in locating acupoints, and corresponds to the distance between the two medial ends of the creases of the interphalangeal joints, when the patient’s middle finger is flexed.

#### Information provided to participants about acupuncture

Participants are informed, during the process for obtaining informed consent, that there is a control group, referred to as a ‘placebo’ group, and that there is a 50% chance of being randomly allocated to this group or the true acupuncture group. All participants are told that the ‘placebo’ needles are designed to stimulate different nerves compared to the true acupuncture needles, and all participants are aware that ‘placebo’ treatment is not considered active treatment. If they specifically enquire, they are told that the acupuncturists remain blinded and receive a pre-specified pack of needles and instructions as to where to insert needles for each participant after randomisation.

### Withdrawal criteria

Participants can withdraw from the trial at any time. The data collected up to the time of withdrawal will be included in the analysis unless participants specifically request for their data to be withdrawn, in which case any unprocessed data will be withdrawn from analysis. This has been made explicit in the Plain Language Statement and Consent Form. Should a participant withdraw, a detailed reason (if provided) is recorded in the trial database.

### Co-interventions

In order to avoid confounding the results of the trial, participants are asked to avoid commencing new pharmaceutical co-interventions for hot flushes during the intervention period, such as herbal treatments and HRT. If already on treatment for hot flushes, participants are requested not to discontinue this treatment during the trial.

### Outcomes

Table 4 summarises the measures and the timing of their collection during the trial.

**Table 4:** Summary of measures collected as part of the Acupause study

		**Run-in**	**Intervention**			**Follow-up**	
**Measure**	**Data collection instrument**	**Baseline**	**First treatment**^**b**^	**4 weeks**	**8 weeks/EOT**^**c**^	**3 months**	**6 months**
Demographic information, risk factor for VMS	Demographics Questionnaire	✓					
VMS frequency, severity and score	Seven-day day flush diary	✓		✓	✓^a^	✓	✓
Health-related Quality of life	Menopause-Specific Quality of life Questionnaire/MENQOL	✓		✓	✓	✓	✓
Depression and anxiety measures	Hospital Anxiety and Depression Scale/HADS	✓		✓	✓	✓	✓
Credibility and expectancy	Credibility Expectancy Questionnaire + additional question on binding		✓				

^a^Primary Outcome Measure. VMS, vasomotor symptoms.

^b^Immediately after the first treatment; ^c^End-of-treatment.

#### Primary outcome measure

The primary outcome measure is the hot flush score at end-of-treatment (EOT). EOT hot flush score is calculated from the seven-day HFD which participants commence immediately upon completing their final treatment. These diaries have been used in multiple trials of interventions, and have been shown to be a consistent and reliable method of evaluating change in VMS [51].

Participants note daily in the diary the number and severity (mild, moderate, severe and very severe) of hot flushes experienced over a twenty-four hour period for seven days. The pilot study showed that it was feasible and acceptable for women to complete the diary over seven days with minimal missing data. The HFD will be used to determine three specific measurements: 1) hot flush frequency; 2) hot flush severity; and 3) a hot flush score comprising both frequency and severity. These scores are calculated as follows:

Hot flush frequency = total number of hot flushes reported/Number of days reported

Hot flush severity = (1 × number of mild hot flushes + 2 × number of moderate hot flushes + 3 × number of severe hot flushes + 4 × number of very severe hot flushes)/Number of hot flushes reported

Hot flush score = (1 × number of mild hot flushes + 2 × number of moderate hot flushes + 3 × number of severe hot flushes + 4 × number of very severe hot flushes)/Number of days reported

#### Secondary outcome measures

i. Hot flush score at other time-points: the primary outcome measure is the hot flush score at the EOT; however, hot flush score will also be measured at four weeks to assess the intermediate effect of acupuncture and at three and six months to measure longer-term outcomes as recommended in the two recent systematic reviews [27,28].

ii. Health related quality of life: the *Menopause-Specific Quality of Life Questionnaire (MENQOL)* is a validated measure of quality of life and clinical change during the menopausal transition and is specific to women in the first eight years post menopause [59]. It consists of 29 items assessing four domains: VMS, physical symptoms, psychological symptoms, and urogenital/sexual symptoms.

iii. Anxiety and Depression: *Hospital Anxiety and Depression Scale (HADS)* is a validated widely-used 14-item self-report scale designed to briefly measure current anxiety and depressive symptoms in non-psychiatric hospital patients. It excludes somatic symptoms, therefore avoiding potential confounding factors [60]. The HADS comprises two independent seven-item subscales for anxiety and depression. Anxiety has been demonstrated to be a predictor of a high placebo response in menopause studies [61].

iv. Expectancy and beliefs about acupuncture: the *Credibility/Expectancy Questionnaire* is a validated, quick and easy-to-administer six-item scale for measuring treatment expectancy and rationale credibility for use in clinical outcome studies [62]. This questionnaire is administered once, immediately after the first treatment.

v. Success of blinding: as surveys to measure the success of blinding may enhance participants’ focus on this, we measure this aspect immediately after the first treatment [63]. There is currently no validated measure to assess blinding in acupuncture research. We will use the scale developed by Bang [64], which ranges from −1 (completely not blinded) to +1 (perfectly blinded).

#### Participants’ characteristics and risk factors for hot flushes

Participants complete a *Demographics Questionnaire* upon enrolment into the trial, which collects demographic information and information on risk factors for hot flushes including:

i. ethnicity [5];

ii. menopausal status - staging system recommended by the Stages of Reproductive Ageing Workshop [65];

iii. smoking status [3];

iv. alcohol use [7,66];

v. physical activity [66];

vi. previous tubal ligation [67];

vii. history of depression [8];

viii. stress levels using the validated Perceived Stress Scale 4 [8,68];

ix. average weekly household income [3,68];

x. education level [68];

xi. BMI [6,7]; and

xii. information on previous use of acupuncture, including types of acupuncture used, date of last treatment, frequency of use, conditions treated, and perceived response to acupuncture

### Data storage and access

All hard copy information that contains personal information on participants or that can be identified is stored in a secure locked cabinet. Electronic data that contains personal information or that can be identified is password-protected on a secure server and can only be accessed by authorised researchers. Information will be retained for a minimum of fifteen years after publication of results.

### Sample size calculation

Sample size calculations are based on a two-sample *t*-test with 80% power and significance level at 5% for a two-sided test. We assumed conservatively that the mean hot flush score at baseline would be 14, the lowest possible entry score for the trial. Assuming a 50% relative reduction in the HF scores in the control group due to the placebo effect, which is the typical response reported in hot flush randomised controlled trials [69], we anticipate that women in the sham acupuncture group will have a mean hot flush score of 7 at EOT. To be clinically significant, we expect that women in the true intervention group to show at least 75% reduction in the total HF score from baseline, with a mean HF score of 3 at EOT. Based on these assumptions, 266 women (133 per trial group) will be needed to detect hot flush score difference of 4 (standard deviation (SD) = 11.6 [51]) between the two trial groups at EOT. This sample size is sufficient to detect a difference of 0.35 of 1 SD between the trial groups for the quality of life measures. Sample size was inflated to 360 women (180 per study group) to allow for attrition of 26% (experienced in the pilot study) at the measurement of the primary outcome at the EOT.

### Randomisation and allocation concealment

#### Sequence generation

Participants are randomly allocated to one of two trial groups: true acupuncture or sham acupuncture, using a computer-generated randomisation sequence, stratified by project acupuncturist using block sizes of either eight or twelve (with block sizes appearing in random order) within each stratum and an allocation ratio of 1:1. The research assistant responsible for allocating participants is not aware of the block sizes used. An independent researcher with no other involvement in the trial used this allocation sequence to create a password-protected electronic ‘spreadsheet’ which contains the covert allocation schedule. The principal investigator (MP) holds the randomisation schedule.

#### Implementation and allocation concealment

Upon confirmation of eligibility of women for the trial (that is, after completion of the baseline HFD and clinical confirmation of diagnosis of Kidney *Yin* deficiency), the unblinded research assistant (KN) uses the spreadsheet to randomly allocate participants to receive either real or sham acupuncture. The spreadsheet displays sequentially only the next treatment allocation.

The research assistant informs the relevant project acupuncturist of the allocation status by mobile phone text messaging, Email or fax, depending on the acupuncturist’s preference. Group allocation is indicated only by the list of acupuncture points for either real or sham acupuncture, to avoid unblinding should the participant inadvertently see the communication. As acupoints used for ‘needling’ in real and sham acupuncture groups are different, acupuncturists can easily identify group allocation from the list of points provided. This process can be cross-checked against the covert randomisation schedule code held by the principal investigator to ensure no corruption of the process has occurred. To prevent selection bias, all baseline information is collected prior to the acupuncturist being informed of the treatment allocation.

### Blinding

The research assistant responsible for randomisation (KN) and the project acupuncturists who deliver the treatment to the women are not blind to treatment allocation. Blinding of project acupuncturists is not possible because of the difference between real and sham acupuncture points used and the tactile difference between real and sham needling.

Participants and all other investigators are blinded, including the study statistician. As outcomes are self-assessed, outcome assessment is blinded. Data analysis will be performed where the treatment groups will be identified as Group A and B. The codes for the treatment groups will be revealed after the all outcome data collection is completed at six months.

### Safety

Prior to providing consent to participate, participants are informed of potential adverse events from acupuncture. Common adverse events include fainting, drowsiness, tiredness, temporary increase in symptoms, bruising and soreness. A practitioner guide to preventing and managing common adverse events is provided in the Practitioner Training Manual. Participants report adverse events either to the project acupuncturist delivering the intervention or the acupuncturist researcher. The project acupuncturist refers to the acupuncturist researcher in the event of ongoing or unresolved concerns. Serious adverse events (defined as potentially life-threatening, permanently incapacitating or resulting in hospitalisation) are notified to the study chief investigator (MP) and the Human Research Ethics Committee within 24 hours. Informed by the pilot project, it is anticipated that telephone advice from a general practitioner (MP/CE) and/or acupuncturist researcher (JS) will be adequate in most cases. If participants require additional medical advice, they are directed to their general practitioner. The expert acupuncturist (CX), who chairs the Chinese Medicine Board in Australia, is also available for advice.

Participants who score higher than 15 on the baseline depression and/or anxiety scales will be contacted by a general practitioner investigator (CE/MP) to be assessed for suicide risk and referred to relevant services as required.

Both the relevant project acupuncturist and the participant complete an Adverse Event Form should the need arise. The form records the dates and times that the event began and resolved; a description of the event; the intensity of the event (mild/moderate/severe); an assessment of the possible relationship to acupuncture, on a four-point Likert scale ranging from ‘unrelated’ to ‘definitely related’; the outcome on a five-point Likert scale ranging from ‘completely resolved’ to ‘persistent’; and whether the event was considered serious (potentially life-threatening, permanent incapacitating or resulting in hospitalisation).

### Data collection

The Credibility/Expectancy Questionnaire, Case Report Form, TCM Diagnosis Form and Adverse Events Form are completed on paper copies. All other outcome measures, participant characteristics and risk factors for hot flushes are completed online using a survey management website (Survey Methods http://www.surveymethods.com), with paper copies available for those who prefer to use them. Online surveys have the advantage of minimising missing data if completion of survey questions is made mandatory prior to submitting the survey online. Range checks are also built into online surveys, and automated reminders are Emailed. The study acupuncturist and research assistant (CE and KN) oversee this data management. A reminder letter/Email is sent after two weeks, followed by a courtesy telephone call two weeks later by the research assistant should questionnaires not be returned. Details of treatment dates and follow-up are recorded in a custom-built password-protected database.

All paper-based data are entered into the same survey management website by staff blinded to group allocation at the University of Melbourne. Ten percent of all data entry will be audited by an independent investigator for quality assurance purposes.

### Statistical methods

Descriptive statistics will be used to summarise baseline measures and participant characteristics between the two trial groups and to assess for chance imbalance of important prognostic factors. Primary outcome is the hot flush score at EOT. Analysis will account for repeated outcome measures taken on the same women over 12 months and stratification by project acupuncturists. Mixed-effects linear regression model will be used to compare the continuous outcomes between the trial groups and marginal logistic regression model using generalised estimating equations with robust standard errors will be used for the binary outcomes. Where appropriate the baseline measure of the outcome will be included as a covariate in the regression model. In addition, factors strongly associated with the outcome and are found to be imbalanced between the trial groups at baseline will also be adjusted in the regression models. Estimates of the intervention effect will be reported as the difference in means between trial groups for continuous outcomes and odds ratios for binary outcomes, with respective 95% confidence intervals and *P*-values. Stata 13.0 [70] will be used for the data analyses.

An intention-to-treat analysis strategy will be employed where all participants will be analysed in the trial group in to which they were allocated [71]. In the first instance, we will strive to minimise the extent of missing outcome data for participants, where all efforts will be made to collect the primary outcome on participants who withdraw, discontinue their treatment or do not respond to the online survey. However, in the presence of incomplete data, information collected on the reasons for missing data will be used to inform the appropriate statistical analysis approach to handle the missing data. Sensitivity analyses that capture departures from the assumption of the missing data mechanism for the primary analysis will also be considered to assess the robustness of the results.

#### Secondary analyses

Additional secondary analyses are planned. These will include (1) exploring the effect acupuncture had on women’s quality of life, (2) identifying factors that are associated with a placebo response, (such as anxiety/depression levels and demographic characteristics such as socioeconomic status), (3) the association between expectancy and beliefs and women’s response to acupuncture treatment, and (4) the credibility of the Park Sham Device.

### Data monitoring

As acupuncture has an excellent safety profile, and is not considered an experimental treatment, no data monitoring committee is required.

## Discussion

Hot flushes are a common and potentially disabling symptom during the menopause. While HRT is an effective treatment, it does not suit all women either because of concerns over potentially serious adverse events, or because of relative contra-indications. Many women also prefer to use natural or complementary therapies where available. There is a paucity of effective and safe non-hormonal treatments for VMS. However, preliminary evidence suggests that acupuncture, a popular and safe treatment for a wide range of conditions, may be effective for VMS.

We are conducting a randomised sham-controlled trial on acupuncture for VMS, which builds on the results from our earlier pilot project during which feasibility of methods was demonstrated. This robustly designed study meets methodological benchmarks of adequate randomisation and allocation concealment procedures, blinding of outcome assessors and statisticians and utilising intention-to-treat analysis strategy. The trial method also allows for Traditional Chinese Medicine diagnosis in the eligibility criteria and a ‘dose’ of acupuncture reflecting clinical practice to ensure results are applicable to a broad range of health practitioners. The trial is appropriately powered to determine a clinically relevant treatment effect. Our results will determine the efficacy of acupuncture for treating VMS in postmenopausal women. In particular, we will assess the specific effect of insertion of the acupuncture needle and its contribution to the entire treatment effect.

Our results will inform women suffering with menopausal symptoms and both conventional and Traditional Chinese Medicine health care professionals on the potential role, if any, of acupuncture in the treatment of VMS. This will advance the field significantly and, if effective, will offer an important treatment option to reduce VMS and potentially improve quality of life in women at this important life stage. Future research may explore the optimal treatment regimen for acupuncture for VMS.

## Trial status

The trial has received funding and recruitment commenced in late 2011. At the time of submission of this protocol, project acupuncturists had been trained, participant enrolment was progressing well and data collection was well underway. Trial enrolment closed in March 2014. We anticipate that all data will be collected by late 2014.

## Abbreviations

BMI: body mass index; CM: complementary medicines; CONSORT: Consolidated Standards of Reporting Trials; EOT: end-of-treatment; FSH: follicular stimulating hormone; HADS: Hospital Anxiety and Depression Scale; HFD: Hot Flush Diary; HRT: hormone replacement therapy; MENQOL: Menopause-specific Quality of Life questionnaire; STRICTA: Standards for reporting interventions in acupuncture trials; RCT: randomised controlled trial; SD: standard deviation; SNRIs: serotonin-noradrenaline reuptake inhibitors; SSRIs: selective serotonin reuptake inhibitors; TCM: Traditional Chinese Medicine; TNZ: thermoneutral zone; VMS: vasomotor symptoms.

## Competing interests

The authors declare that they have no competing interests.

## Authors’ contributions

CE conceived of the idea and designed the pilot project with input from MP and CX. CE, MP, HT, CX, PC, SM and SF contributed to the design of the RCT. CE and MP wrote the first draft of the manuscript. All authors have contributed to, read and approved the final manuscript.

## Authors’ information

MP - MS BS, M Med, FRACGP, DRANZCOG, PhD. CE - MB BS, B.Appl.Sci (Human Bio/Chinese Medicine), M Med, FRACGP, GradCertMedAcup. HT - MBBS, FRACP, PhD. PC - B Sc, GradDipEpi&Biostat, MSc(Stat), PhD. SF - BAppSc, MPH, PhD. SM - PhD BMed ND. CX - BMed, PhD.
