# Erratum to: Acupuncture for menopausal vasomotor symptoms: study protocol for a randomised controlled trial

**DOI:** 10.1186/s13063-015-0596-2

**Published:** 2015-03-12

**Authors:** Marie Pirotta, Carolyn Ee, Helena Teede, Patty Chondros, Simon French, Stephen Myers, Charlie Xue

**Affiliations:** Department of General Practice, University of Melbourne, 200 Berkeley St, Carlton, 3053 Victoria Australia; Monash Centre for Health Research and Implementation, School of Public Health and Preventive Medicine, Monash University, Melbourne, Australia; Diabetes and Vascular Medicine Unit, Monash Health, Melbourne, Australia; School of Rehabilitation Therapy, Queen’s University, Ontario, Canada; Faculty of Health Sciences, Ontario, Canada; Division of Research, NatMed-Research Unit, Southern Cross University, Lismore, Australia; School of Health Sciences, RMIT University, Melbourne, Australia

## Erratum

After the publication of this work [1], it was brought to our attention that the time for patient follow-up specified in the ‘Statistical methods’ section of the protocol was incorrect; follow-up will continue for six months after the end of treatment, not 12 months as stated in the original published version.
